# Contralateral efferent suppression of human hearing sensitivity

**DOI:** 10.3389/fnsys.2014.00251

**Published:** 2015-01-15

**Authors:** Enzo Aguilar, Peter T. Johannesen, Enrique A. Lopez-Poveda

**Affiliations:** ^1^Auditory Computation and Psychoacoustics, Instituto de Neurociencias de Castilla y León, Universidad de SalamancaSalamanca, Spain; ^2^Grupo de Audiología, Instituto de Investigación Biomédica de Salamanca, Universidad de SalamancaSalamanca, Spain; ^3^Departamento de Cirugía, Facultad de Medicina, Universidad de SalamancaSalamanca, Spain

**Keywords:** temporal integration, absolute threshold, auditory efferents, audiometry, central masking, auditory models, auditory suppression

## Abstract

The present study aimed at characterizing the suppressing effect of contralateral medial olivocochlear (MOC) efferents on human auditory sensitivity and mechanical cochlear responses at sound levels near behavioral thresholds. Absolute thresholds for pure tones of 500 and 4000 Hz with durations between 10–500 ms were measured in the presence and in the absence of a contralateral broadband noise. The intensity of the noise was fixed at 60 dB SPL to evoke the contralateral MOC reflex without evoking the middle-ear muscle reflex. In agreement with previously reported findings, thresholds measured without the contralateral noise decreased with increasing tone duration, and the rate of decrease was faster at 500 than at 4000 Hz. Contralateral stimulation increased thresholds by 1.07 and 1.72 dB at 500 and 4000 Hz, respectively. The mean increase (1.4 dB) just missed statistical significance (*p* = 0.08). Importantly, the across-frequency mean threshold increase was significantly greater for long than for short probes. This effect was more obvious at 4000 Hz than at 500 Hz. Assuming that thresholds depend on the MOC-dependent cochlear mechanical response followed by an MOC-independent, post-mechanical detection mechanism, the present results at 4000 Hz suggest that MOC efferent activation suppresses cochlear mechanical responses more at lower than at higher intensities across the range of intensities near threshold, while the results at 500 Hz suggest comparable mechanical suppression across the threshold intensity range. The results are discussed in the context of central masking and of auditory models of efferent suppression of cochlear mechanical responses.

## Introduction

Physiological studies in non-human mammals have shown that activation of olivocochlear efferents suppresses cochlear mechanical responses. The amount of suppression is greater at low than at moderate or high sound input levels (Murugasu and Russell, 1996; Dolan et al., 1997; Russell and Murugasu, 1997; Cooper and Guinan, 2006; Guinan and Cooper, 2008). Olivocochlear efferents may be activated in a reflexive manner by contralateral stimulation (Guinan, 2006, 2010). On the other hand, otoacoustic emissions (OAEs) are a byproduct of cochlear mechanical responses (Kemp, 1978, 2002). For these reasons, researchers have often looked at the suppression of OAEs by contralateral stimulation as a way to physiologically characterize the suppressing effects of the contralateral medial olivocochlear reflex (MOCR) in humans (Guinan et al., 2003; Atcherson et al., 2008; Sun, 2008; Lilaonitkul and Guinan, 2009a,b; Francis and Guinan, 2010). Comparatively, few studies have looked at the suppressing effects of the contralateral MOCR on human auditory sensitivity. This is the aim of the present study.

Typically, auditory sensitivity is assessed by measuring the detection threshold of pure tones. It has been long known that pure-tone detection thresholds increase with contralateral stimulation (Wegel and Lane, 1924). Contralateral pure tones increase thresholds between 3 and 15 dB, depending on the frequency and the temporal position of the test tone relative to that of the contralateral tone. Typically, threshold increases are larger for test tones at the frequency and at the onset of the contralateral tone than in other conditions (Zwislocki et al., 1967, 1968; Mills et al., 1996). This phenomenon was originally referred to as “central masking” because the contralateral stimulus was regarded as a “masker” and the threshold increase was interpreted to occur by interaction of that “masker” with the test tone somewhere in the central auditory nervous system (Zwislocki et al., 1967).

Smith et al. (2000) related central masking with MOC suppression. They used macaques and measured absolute thresholds for pure tones of 1 s of duration in the presence and in the absence of a continuous, band-limited (two-octave wide) contralateral noise centered at the test tone frequency. They reported mean threshold shifts of 2, 4 and 6 dB at 1, 2, and 4 kHz, respectively, for a contralateral noise at 60 dB of sensation level (SL). Most importantly, they showed that the shift decreased in magnitude, or even disappeared, after sectioning of MOC efferents. Hence, they argued that central masking is probably due to the suppressing effects of the MOCR and concluded that the term “central masking” is probably incorrect.

Kawase et al. (2003) measured the suppressor effect of the contralateral MOCR on audiometric thresholds for pure tones at frequencies of 500–8000 Hz. They used a continuous broadband noise as the contralateral MOCR elicitor and their test tones had 50 ms of duration. They showed that their contralateral noise increased audiometric thresholds more at low than at high frequencies (see their Figure 2), and that the magnitude of the increase was proportional to the intensity of the contralateral stimulus. Their results are broadly consistent with more recent studies reporting greater OAE suppression at low than at high frequencies (Lilaonitkul and Guinan, 2009a).

Human auditory sensitivity not only depends on the frequency of the test tone; it also depends on its duration. Absolute thresholds decrease with increasing tone duration and the rate of decrease varies depending on tone frequency (Watson and Gengel, 1969). Several explanatory mechanisms have been proposed for this phenomenon, including the idea that threshold is based on the integration of sound intensity (Watson and Gengel, 1969) or sound pressure (Heil and Neubauer, 2003) over time, on taking “multiple-looks” at neural responses elicited by the test tones in search for excess neural activity (Viemeister and Wakefield, 1991), or on the greater probability of firing of auditory nerve fibers for longer than for shorter stimuli (Meddis, 2006).

**Figure 1:**
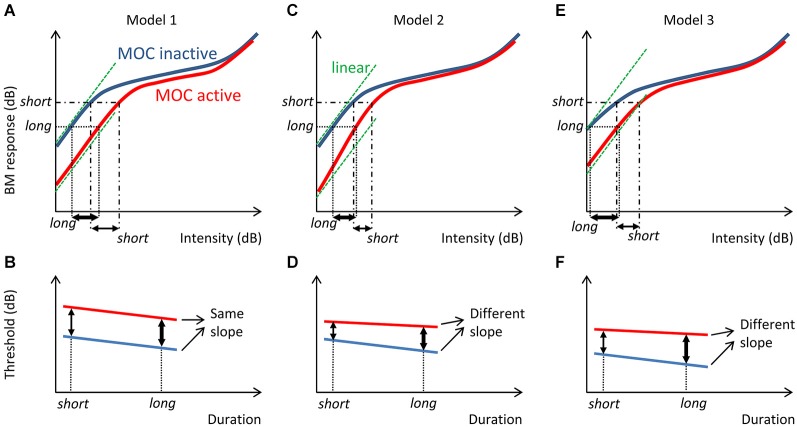
**Three possible models of contralateral MOC suppression of basilar membrane (BM)** responses and their effects on threshold-vs.-duration functions. The left panels (Model 1) illustrate the case where BM responses are linear (slope = 1 dB/dB) at behavioral threshold both with and without MOC activation and MOC activation suppresses BM responses by a constant amount at all levels near threshold, a model adapted from (Ferry and Meddis, 2007). Assuming that a short tone requires a greater BM response than a longer tone to evoke just detectable responses **(A)**, MOC activation would vertically shift the threshold-vs.-duration function without changing the function slope **(B)**. The middle panels (Model 2) illustrate an alternative model where BM responses are linear at threshold and MOC activation suppresses BM responses more at low than at moderate input threshold levels **(C)**. In other words, in this case MOC activation would turn a linear BM input/output function near threshold into expansive (slope > 1 dB/dB). In this case, MOC activation would vertically shift the threshold-vs.-duration function with a concomitant change in slope **(D)**. The right panels illustrate a third model (Model 3) where BM responses are slightly compressive (slope < 1 dB/dB) at threshold and MOC activation suppresses BM responses more at low than at moderate input levels in the threshold range, thus causing the compressive BM input/output curve to become linear near threshold **(E)**. In this case, MOC activation would also vertically shift the threshold-vs.-duration function with a concomitant change in slope **(F)**. For reference, the green dashed lines illustrate hypothetical linear input/output relationships.

**Figure 2:**
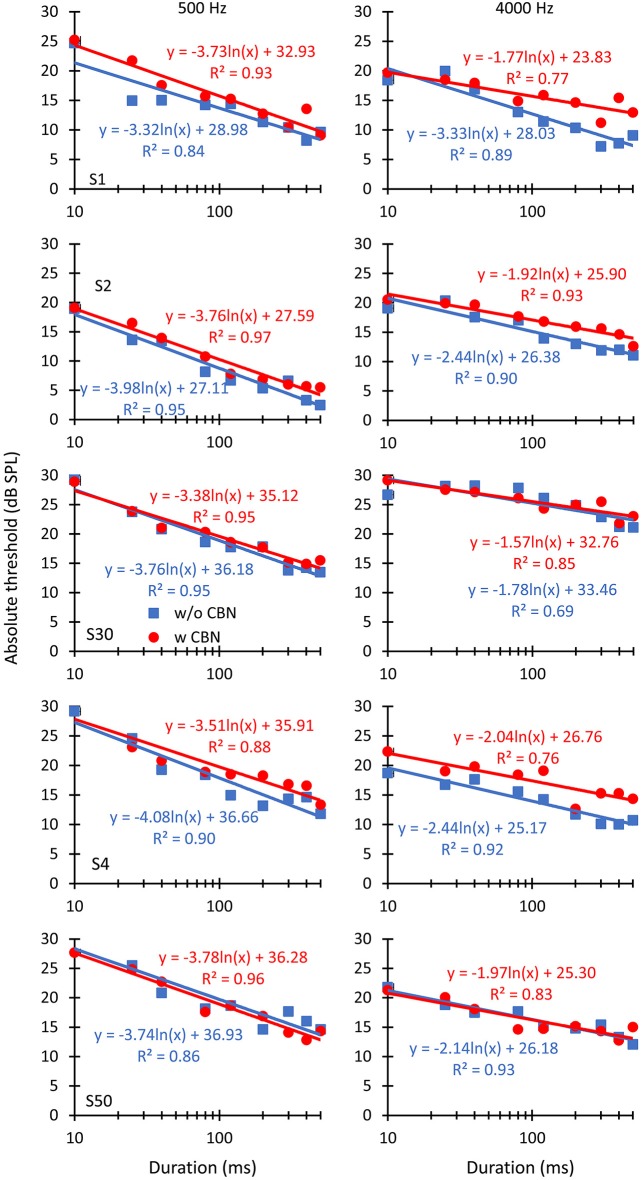
**The effect of contralateral stimulation on individual absolute detection thresholds for pure tones of different durations**. The left and right columns illustrate results for pure tone frequencies of 500 and 4000 Hz, respectively. Each row illustrates results for an individual listener, as indicated in the left panels. Symbols depict mean thresholds in the absence (“w/o CBN”, squares) and in the presence (“w CBN”, circles) of a CBN, as indicated by the inset in the left panel of the middle row. Lines illustrate least-squares fits to the data using the logarithmic functions given next to the data. *R*^2^ is the proportion of variance in the data predicted by the fits.

Whatever the actual mechanism, threshold probably depends on the magnitude of the cochlear mechanical response elicited by the stimulus and a post-mechanical detection mechanism. In the absence of efferent suppression, human mechanical cochlear responses are probably linear at intensities near absolute threshold (Plack and Skeels, 2007). Assuming that the post-cochlear detection mechanism is independent of MOC activation, comparisons of the threshold-vs.-duration functions measured with and without contralateral stimulation can give us some insight about the magnitude of MOC suppression of basilar membrane (BM) responses at different sound levels near threshold. For example, if the amount of suppression were comparable across levels near threshold, as is illustrated by Model 1 in Figure 1, MOC activation would shift the threshold-vs.-duration function vertically without a change in the function slope. By contrast, if the magnitude of MOC suppression were greater at lower than at higher sound levels (throughout the threshold range), as is illustrated by Model 2 in Figure 1, or if BM responses were compressive at threshold for short tones and linearized by MOC suppression, as illustrated by Model 3 in Figure 2, then MOC activation would shift the threshold-vs.-duration function with a concomitant change in slope. The aim of the present study was to test these hypotheses by comparing threshold-vs.-duration functions measured in the presence and in the absence of a 60-dB SPL contralateral broadband noise (CBN) used as the MOCR elicitor.

## Methods

### Stimuli

The task consisted of measuring absolute detection thresholds of pure tones of various different durations in the presence and in the absence of a CBN used as the MOCR elicitor. Pure tones had frequencies of 500 and 4000 Hz, and durations of 10, 25, 40, 80, 120, 200, 300, 400, and 500 ms. All of them were gated with onset and offset raised-cosine ramps of 5-ms of duration. The level of the CBN was set fixed at 60 dB SPL. It has been shown elsewhere that this type of noise is capable of activating the MOCR without activating the middle-ear muscle reflex (Lilaonitkul and Guinan, 2009a,b; Aguilar et al., 2013). The CBN had a duration of 1010 ms and it was gated with 5-ms raised-cosine onset and offset ramps. It started 500 ms before the tone onset and ended 10 ms after the offset of the longest tone. The MOCR is almost fully activated about 330 ms after the elicitor onset (Backus and Guinan, 2006). Therefore, we assumed that MOCR was activated at the onset of the test tone and remained active over the whole tone duration.

### Procedure

Absolute detection thresholds were measured using a two-interval, two-alternative, forced-choice adaptive procedure. Two intervals were presented to the participants accompanied by flash lights in a computer monitor. One of the intervals was silent in the test ear while the other contained the test tone; the CBN was presented to the contralateral ear in the two intervals. The inter-stimulus time interval (defined as the silent period between the offset and the onset of the CBN in the two intervals) was 500 ms. The test tone was presented in either the first or the second interval at random and participants were instructed to identify the interval containing the tone by pressing a key on the computer keyboard. Feedback was given to the participants. The level of the test tone decreased after two successive correct responses and increased after an incorrect response (two-down, one-up adaptive rule). Absolute threshold was thus defined as the tone level giving 71% correct responses in the psychometric function (Levitt, 1971). The level of test tone changed by 6 dB until the third reversal in level occurred, and it changed by 2 dB thereafter. The procedure continued until 12 level reversals were measured and absolute threshold was obtained as the mean of the tone levels at the last 10 reversals. The threshold estimate was discarded when the corresponding standard deviation exceeded 6 dB. At least three valid thresholds estimates were obtained for each condition and their average was taken as the absolute threshold. When the standard deviation of the three threshold estimates exceeded 6 dB, additional estimates were obtained and included in the mean.

Stimuli were generated with custom-made Matlab software and played via an RME Fireface 400 soundcard at a sampling rate of 44.1 kHz, and with 24-bit resolution. Stimuli were presented to the participants using Etymotic ER-2 insert earphones. These earphones are designed to give a flat frequency response at the eardrum and have a nominal inter-aural attenuation of 70+ dB that minimizes cross-hearing. Listeners sat in a double-wall sound attenuating booth during all measurements.

Stimuli were calibrated by coupling the earphones to a sound level meter (B&K 2238) through a Zwislocki coupler (Knowles DB-100). Calibration was performed at 1 kHz and the measured sensitivity was applied to all other frequencies.

Experimental procedures were approved by the Human Experimentation Ethics Committee of the University of Salamanca (Spain). Informed consent was obtained from all participants.

### Subjects

Three women (S1, S4, and S50) and two men (S2—the first author, and S30) with no history of hearing impairment participated in the study. All of them had normal tympanometry and clinical audiometric thresholds within 20 dB hearing level (HL; ANSI, 1996). Their ages were 31 (S1), 31 (S2), 27 (S4), 33 (S30), and 25 (S50) years. Subjects were volunteers and were not paid for their service.

## Results

### Absolute thresholds

**Figure 3:**
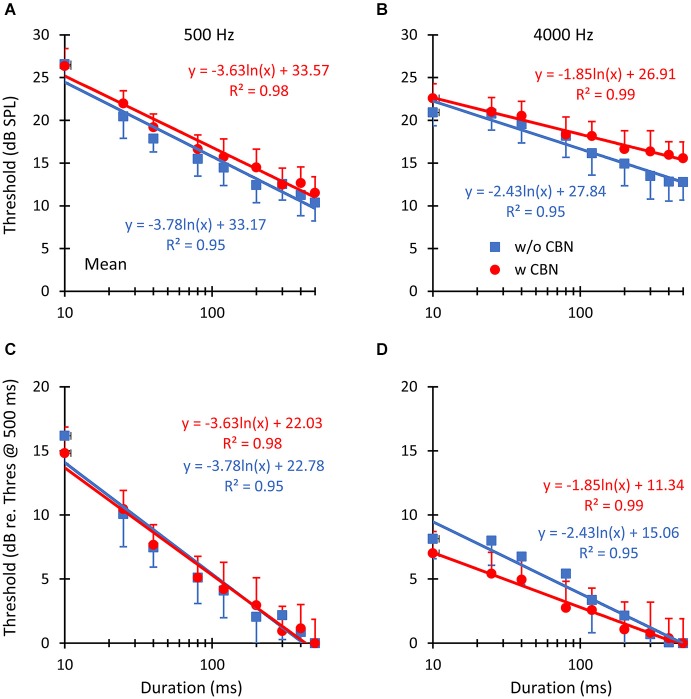
**The *mean* effect of contralateral stimulation on absolute detection thresholds for pure tones of different durations**. The left and right columns illustrate results for pure tone frequencies of 500 and 4000 Hz, respectively. Symbols depict mean thresholds in the absence (“w/o CBN”, squares) and in the presence (“w CBN”, circles) of a CBN, as indicated by the inset in panel B. Error bars illustrate one standard error of the mean. Lines illustrate least-squares fits to the data using the logarithmic functions given next to the data. The two rows show identical data in units of dB SPL **(A,B)** or dB re threshold for the 500-ms tone **(C,D)**.

Individual and mean threshold-vs.-duration functions are shown in Figures 2, 3, respectively. A first question is whether the CBN increases absolute thresholds, as would be expected (e.g., Figure 1), and whether the magnitude of the increase is different at the two probe frequencies or for different probe durations. Visual inspection of Figures 2, 3 suggests that the CBN raised thresholds at the two test frequencies for some listeners (S1, S2 or S4) but not for others (S30 and S50). When it occurred, the threshold increase was typically greater for long than for short durations, particularly at 4000 Hz. A three-way repeated-measures analysis of the variance (ANOVA) was used to test for the effects of duration, probe frequency, the presence of contralateral stimulation, and their possible interactions on mean absolute thresholds. Results revealed a statistically significant interaction between duration and frequency (*F*_(8,32)_ = 29.55, *p* < 0.001). All other possible interactions between pairs of factors or between the three factors were not statistically significant. The analysis also revealed a significant effect of duration (*F*_(8,32)_ = 160.87, *p* < 0.001) and an almost-significant effect of contralateral stimulation (*F*_(1,4)_ = 5.64, *p* = 0.080) but not a significant effect of frequency.

**Figure 4:**
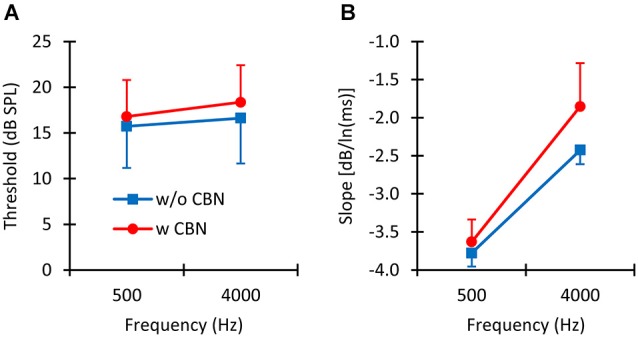
**The mean effect of contralateral stimulation. (A)** Mean absolute detection thresholds with (w CBN) and without (w/o CBN) contralateral stimulation at the two test frequencies. **(B)** Mean slope of the threshold-vs.-duration functions with and without contralateral stimulation at the two test frequencies. Error bars illustrate one standard deviation.

In summary, thresholds were: (1) lower for longer than for shorter probes (Figure 3); and (2) higher in the presence than in the absence of the CBN (Figure 4A), although the effect of the CBN just missed statistical significance. On average, the CBN increased thresholds slightly more at 4000 than at 500 Hz (1.72 vs. 1.07 dB, respectively), but the effect of frequency on the mean increase was not statistically significant.

### Slope of the threshold-vs.-duration function

Figures 2, 3 show that in the absence of contralateral stimulation, absolute thresholds always decreased with increasing tone duration and the rate of decrease (i.e., the slope of the function) generally appeared steeper at 500 than at 4000 Hz. These results are consistent with those reported by early studies (Watson and Gengel, 1969). The question here is whether the contralateral stimulation alters the slope of the threshold-vs.-duration function and if so, whether the change is different at the two test frequencies. To better address this question, logarithmic functions were fitted *ad hoc* to the individual data. The fits are shown in Figures 2, 3 as straight lines together with their corresponding equations and goodness-of-fit statistic, *R*^2^ (the proportion of predicted variance). The high values of *R*^2^ support the chosen model.

Figure 4B illustrates the effect of the CBN on the mean slope at the two test frequencies. Table 1 gives corresponding numeric values. A two-way repeated-measures ANOVA was carried out to test for the effects of frequency, the presence or absence of contralateral stimulation, and their possible interaction on the *slope* of the threshold-vs.-duration function. The test revealed that when the data for the two CBN conditions were combined, mean slopes were significantly steeper at 500 than at 4000 Hz: mean slopes were −3.70 vs. −2.14 dB/ln(ms) at 500 and 4000 Hz respectively (*F*_(1,4)_ = 99.37, *p* = 0.001). The test also revealed that when the data for the two frequencies were combined, mean slopes were significantly shallower with than without contralateral stimulation: mean slopes were −2.74 vs. −3.10 dB/ln(ms) with and without CBN, respectively (*F*_(1,4)_ = 16.73, *p* = 0.015). Figure 4B shows that the CBN reduced the mean slope more at 4000 than at 500 Hz (see also Figures 3C,D). Although the ANOVA revealed that this effect (i.e., the interaction of frequency with CBN effects on the slope) was not statistically significant (*F*_(1,4)_ = 1.16, *p* = 0.342), a *post hoc* analysis suggested that the mean slope decrease (Table 1) was almost significant at 4000 Hz (two-tailed paired *t*-test, *N* = 5, *p* = 0.088) and not significant at 500 Hz (two-tailed, paired *t* test, *N* = 5, *p* = 0.446).

**Table 1:** **Slope (in units of dB/ln(ms)) of the threshold-vs.-duration functions**.

	w/o CBN	w CBN	Mean
500 Hz	−3.77 ± 0.29	−3.63 ± 0.18	−3.70
4000 Hz	−2.43 ± 0.57	−1.85 ± 0.19	−2.14
Mean	−3.10	−2.74

*Central cells give mean of the individual slopes across the five listeners plus and minus one standard deviation*.

In summary, the rate of decrease of absolute threshold with increasing duration (i.e., the slope of the threshold-vs.-duration function) was (1) faster at 500 than at 4000 Hz; and (2) decreased in the presence of a CBN. The CBN reduced the rate of decrease more at 4000 Hz than at 500 Hz (Table 1). At 4000 Hz, the reduction in slope just missed statistical significance.

## Discussion

The aim of the present study was to investigate the effect of MOC activation on the detection threshold of pure tones of different durations. We used a CBN with a level of 60 dB SPL as the MOCR elicitor because it has been shown in previous reports that this noise appears sufficient to evoke an MOCR without activating a middle-ear muscle reflex (see Figure 1 in Aguilar et al., 2013; see also Lilaonitkul and Guinan, 2009a). Therefore, it is reasonable to assume that the reported effects of the CBN are caused by the activation of contralateral MOC efferents.

We have shown that in the absence of contralateral acoustic stimulation, (1) pure-tone absolute thresholds decrease with increasing tone duration; and (2) that the rate of decrease is steeper at 500 Hz than at 4000 Hz (compare the slope of the blue lines in Figures 3C,D; see also Table 1). This aspect of the results is not new and is broadly consistent with what was shown by Watson and Gengel (1969).

We have also shown that the CBN employed here increases absolute thresholds on average by 1.07 and 1.72 dB at 500 and 4000 Hz, respectively (Figure 4A), presumably by activation of the MOCR. (We note, however, that the increase just missed statistical significance (*p* = 0.08) because the effect of the CBN was largely variable across listeners (Figure 2)). It is hard to compare this result with conclusions from previous studies because the magnitude of MOC suppression depends on the intensity and time course of the MOC elicitor in relation to the probe (Zwislocki et al., 1967; Kawase et al., 2003). Even studies that used an MOCR elicitor comparable to the one employed here show mixed effects, not always consistent with the present data. For example, Lilaonitkul and Guinan (2009a) showed that a contralateral MOCR elicitor suppressed human stimulus frequency OAEs more at 500 than at 4000 Hz (their Figure 3). Smith et al. (2000) reported that for macaques, the contralateral MOCR elicitor increased threshold shifts more at 4000 than at 500 Hz (their Figure 2). Kawase et al. (2003) showed that the MOCR elicitor shifted absolute thresholds by approximately equal amounts at the two frequencies, but the magnitude of the shift was around 6 dB, hence greater than the magnitude observed in the present data (1.4 dB). Aguilar et al. (2013) analyzed the effect of the CBN on psychoacoustical tuning curves and concluded that suppression was greater at 500 than at 4 kHz. The reason for the discrepancies across studies is uncertain. It might be related to differences in experimental species, procedures, and/or MOCR elicitor-probe timing configurations. Indeed, previous studies on the effect of the MOCR on OAEs or psychoacoustical tuning curves were concerned with effects at supra-threshold conditions while the present study focuses on effects at absolute threshold.

The main novelty of the present results is that *on average* the magnitude of threshold increase caused by the CBN was greater for long than for short probe durations; in other words, that the CBN made the threshold-vs.-duration functions shallower (Figure 4B). This effect was more obvious at 4000 Hz than at 500 Hz (Figures 3C,D and Table 1), even though in statistical terms the effect was not significantly different at the two frequencies. Assuming that threshold shifts are caused by a reduction of mechanical cochlear responses only (i.e., that MOC activation does not affect post-mechanical detection mechanism) and based on the rationale illustrated in Figure 1, the present results suggests that at 4000 Hz MOC activation reduces cochlear mechanical responses more at lower than at higher input levels in the threshold range, while at 500 Hz the reduction of cochlear mechanical responses is comparable at all levels in the threshold range. In other words, the present results at 500 Hz appear consistent with Model 1 in Figure 1, while the results at 4000 Hz appear consistent with either Model 2 or Model 3.

Model 1, however, has been successfully used elsewhere to mimic the effects of MOC activation on the vibration of basal BM regions and on the spike rate of auditory nerve fibers with high characteristic frequencies (Ferry and Meddis, 2007). It has also been successfully used to model the effects of the contralateral MOCR on psychoacoustical tuning curves at 500 and 4000 Hz (Aguilar et al., 2013; Lopez-Poveda et al., 2013). That the present data at 4000 Hz are *not* consistent with such model is thus puzzling and the reason uncertain. One possibility is that in testing Model 1, previous studies put more emphasis in getting accurate model predictions at levels above around 30 dB SPL than at the lower levels measured here. Indeed, Figure 2 in Ferry and Meddis (2007) reveals that Model 1 is comparatively less accurate at mimicking the efferent effects on BM input/output curves at low than at moderate input levels.

On the other hand, Model 2 assumes that linear BM responses at threshold become *expansive* with MOC efferent activation, something not supported by experimental BM input/output curves from basal cochlear regions. Instead, direct recordings of BM motion from basal cochlear regions suggest that BM input/output curves are slightly compressive near behavioral thresholds and become more linear with MOC efferent activation (e.g., Figure 2 in Cooper and Guinan (2006); Figure 2 in Murugasu and Russell (1996)). In other words, physiological recordings in non-human mammals suggest that the present human data at 4000 Hz would be consistent with Model 3.

That the effect of MOC activation on the slope of the threshold-vs.-duration function was not significantly different at the two test frequencies was possibly due to the large variability of the data across subjects (Figures 2, 4B). We note, however, that the effect of the CBN on the slope was almost statistically significant at 4000 Hz (*p* = 0.088) but far from significant at 500 Hz. The available mechanical measurements indicate considerable differences between cochlear mechanics in the apex vs. in the base (Robles and Ruggero, 2001; Cooper, 2004). Therefore, although admittedly uncertain, it is conceivable that the more obvious MOC effects at 4000 than at 500 Hz do reflect actual differences in cochlear mechanics between basal and apical cochlear regions.

One might think that the comparatively larger threshold increase for longer tones could be due to inhibition from the ipsilateral MOCR that would be evoked by the longer test tones and less so by the shorter tones. This, however, is unlikely because all the tones involved in the present study had levels at behavioral threshold, and tones at threshold elicit little or no MOC activity.

The threshold shifts reported here are interpreted to be primarily caused by suppression of cochlear mechanical responses by MOC efferent activation. Electrically stimulated ears lack contralateral MOC efferent modulation of cochlear mechanical responses and yet contralateral masking has been reported to occur on electrically stimulated ears of cochlear implant users with residual hearing in the non-implanted ear (James et al., 2001) as well as in bilateral cochlear implant users (Lin et al., 2013). Therefore, it is conceivable that central masking does occur in spite of the evidence given by Smith et al. (2000) and that it may have contributed to the present results to some uncertain extent.

## Conclusions

1. Activation of the contralateral MOCR by broadband noise at 60 dB SPL increased the absolute detection thresholds of pure tones for some listeners but not for others. The average increase was 1.07 and 1.72 dB at 500 and 4000 Hz, respectively, and just missed statistical significance because of the wide across-subject variability.
2. On average, the threshold increase due to MOCR activation was significantly greater for longer (500 ms) than for shorter (10 ms) probes. This effect was more obvious at 4000 than at 500 Hz.
3. Assuming that detection thresholds depend on the MOC-dependent cochlear mechanical response followed by an MOC-independent, post-mechanical detection mechanism, the present results at 4000 Hz suggest that MOC efferent activation suppresses cochlear mechanical responses more at lower than at higher intensities across the range of intensities near threshold, while the results at 500 Hz suggest that MOC efferent activation suppresses cochlear mechanical responses similarly across the range of intensities near threshold. In other words, the present results would be consistent with Model 1 in Figure 1 at 500 Hz and with Model 3 at 4000 Hz.

## Conflict of interest statement

The authors declare that the research was conducted in the absence of any commercial or financial relationships that could be construed as a potential conflict of interest.
